# Seeing others is believing – Analgetische Placeboeffekte durch Beobachtungslernen?

**DOI:** 10.1007/s00482-022-00646-w

**Published:** 2022-04-13

**Authors:** Marie Schwartz, J. Stuhlreyer, R. Klinger

**Affiliations:** Klinik für Anästhesiologie, Schmerzmedizin und Schmerzpsychologie, Universitätsklinikum Hamburg-Eppendorf, Martinistr. 52, 20246 Hamburg, Deutschland

**Keywords:** Schmerz, Übersichtsarbeit, Soziales Lernen, Beobachtungslernen, Erwartung, Pain, Review, Social learning, Observational learning, Expectation

## Abstract

**Hintergrund:**

Es gibt viele Studien zur Placeboanalgesie und deren zugrundliegenden Wirkmechanismen, die eine Behandlung von Patient:innen mit chronischen Schmerzen signifikant verbessern können. Beobachtungslernen als ein Wirkmechanismus wurde hingegen noch wenig untersucht.

**Fragestellung:**

Das Ziel der Arbeit ist es, einen Überblick über die aktuelle Forschungslage zu Placeboanalgesie durch Beobachtungslernen zu geben. Dabei soll geklärt werden, ob Beobachtungslernen überhaupt einen signifikanten Placeboeffekt auslösen kann und durch welche Faktoren dieses Lernen beeinflusst wird.

**Material und Methoden:**

Dafür wurden die Forschungsdatenbanken nach Studien zur Placeboanalgesie durch Beobachtungslernen durchsucht.

**Ergebnisse:**

Nach der Anwendung der Ein- und Ausschlusskriterien verblieben 12 Studien. Es gab nur eine Studie, die an Patient:innen mit chronischen Schmerzen durchgeführt wurde. Die geringe Anzahl an Studien lässt noch keine allgemeingültigen Aussagen zu, aber es gibt erste Hinweise für die folgenden Aussagen: Beobachtungslernen von Placeboeffekten ist unter Laborbedingungen möglich und eine Aufmerksamkeitslenkung ist wichtig. Die Effektstärken reichen von klein bis groß. Die Effekte von klassischer Konditionierung und Beobachtungslernen sind vergleichbar. Live-Modelle, Videoaufnahmen und Bilder lösen ähnliche Effekte aus. Beobachtungslernen führt zu einer Erwartungsänderung.

**Diskussion:**

Die vorliegende Evidenz liefert die Grundlage dafür, dass theoretisch und auch praktisch klinisch signifikante Effekte möglich sind. Weitere Studien sind nötig, um diese Aussagen verlässlich auch auf chronische Schmerzpatient:innen zu beziehen.

## Abstract (en)

**Background:**

There are many studies on placebo analgesia and its underlying mechanisms that show a significant improvement of care for chronic pain patients. However, observational learning has not been researched to this degree.

**Objectives:**

The goal of this work is to provide an overview of the research on placebo analgesia via observational learning. The evidence around whether observational learning can induce placebo analgesia will be discussed. Also, research on the factors that influence observational learning of placebo analgesia will be reviewed.

**Materials and methods:**

To this end, research data bases were searched for studies on placebo analgesia via observational learning.

**Results:**

After inclusion and exclusion criteria were implemented, 12 studies remained. To date, there has been only one study with patients with chronic pain. The small number of included studies do not permit universal statements. However, there is preliminary evidence that observation triggers placebo analgesia as an independent mechanism. Observational learning in an experimental setting can induce placebo effects. Attention focusing on the observation might be critical. The effect sizes tend to be small to large. The effect of classical conditioning and observational learning seem to be of equal size. Live models, video recordings and even pictures of models also induce similar effects. Observational learning induces a change in expectation.

**Discussion:**

The evidence included provides the theoretical basis for potential significant clinical impact. Further research is needed to extend these findings to chronic pain patients.

## Hintergrund

Beobachtet eine Person, wie ein anderer Patient oder eine andere Patientin von einer Schmerztherapie profitiert, dann kann dies entscheidend beeinflussen, wie sie selber von dieser Schmerztherapie profitiert. Der Grund hierfür liegt darin, dass Schmerzen abhängig von ihrem Kontext [5], in dem sie erlebt werden, unterschiedlich stark wahrgenommen werden können und damit auch deren Behandlung sehr stark durch den Kontext, in dem sie umgesetzt wird, beeinflusst ist. Die Beobachtung, dass der:die Bettnachbar:in im Krankenhaus durch eine Infusion deutlich schmerzgelindert ist, kann bei den Beobachtenden die Erwartung aufbauen, dass diese Infusion auch bei ihnen eine positive Wirkung erzielt. Diese Erwartung formt den Kontext für die Behandlung: Erhalten die Beobachtenden in Folge genau die gleiche Behandlung wie der:die Bettnachbar:in, dann scheint eine effektive Schmerzreduktion wahrscheinlicher zu sein.

Für die Erforschung der Kontextabhängigkeit des Schmerzes wurde das Modell des analgetischen Placeboeffekts und auch des algetischen Noceboeffekts entwickelt. Durch diesen Forschungsbereich wurden wesentliche Beiträge zum Verständnis der Modulation von Schmerzen durch psychische Einflussfaktoren geleistet [10, 16]. Der analgetische Placeboeffekt beschreibt eine Schmerzlinderung nach Einnahme z. B. einer Substanz ohne spezifischen Wirkstoff, des sogenannten Placebos. Der Noceboeffekt hingegen beschreibt eine Verschlechterung nach der Einnahme einer Substanz ohne spezifischen Inhaltsstoff, des Nocebos. Eine klinisch relevante Anwendung von Placeboeffekten ist es, diese als Additiv zur Wirksamkeit von „echten“ Medikamenten oder Behandlungen anzuwenden. Insofern ist es wichtig, die Entstehungsmechanismen von Placeboeffekten zu erforschen, um diese gezielt aufbauen und optimieren zu können. Umgekehrt ist dies auf den Noceboeffekt zu übertragen. Die Kenntnis der Entstehungsmechanismen kann helfen, Noceboeffekte bei Behandlung zu verhindern.

Erwiesene Wirkmechanismen des Placeboeffekts sind klassische Konditionierung und verbale Instruktion

Als zugrundeliegende Wirkmechanismen des Placeboeffekts konnten die klassische Konditionierung und verbale Instruktion nachgewiesen werden [10, 16]. Beide haben einen maßgeblichen Einfluss auf die Erwartung von Patienten und sind als grundlegende modulierende Einflussvariablen für analgetische Placeboeffekte identifiziert worden [6]. Daneben wird seit einiger Zeit [9] auch das Beobachtungslernen als möglicher Wirkmechanismus des Placebo- und Noceboeffekts diskutiert.

Bei der klassischen Konditionierung handelt es sich um die Koppelung eines unkonditionierten Stimulus (z. B. Futternapf) und der unkonditionierten Reaktion (z. B. Speichelfluss) mit einem neutralen Stimulus (z. B. Glocke). Dies führt zu einer konditionierten Reaktion auf den nun konditionierten Stimulus (vorher neutraler Stimulus). Die verbale Instruktion bezeichnet eine Informationsvermittlung durch verbale Mitteilungen oder Suggestionen (z. B. „Bei dem grünen Licht werden Sie weniger Schmerzen spüren.“). Auch der Einfluss des sozialen Kontextes der Behandlung wurde vielfach untersucht [12, 13, 19]. Das Beobachtungslernen als zugrundeliegender Mechanismus des Placeboeffekts und als Einflussfaktor auf Behandlungserwartungen von Schmerzpatienten wurde jedoch noch nicht umfassend untersucht, insbesondere fehlen klinische Studien zu diesem Thema. Der vorliegende Artikel befasst sich aus diesem Grunde mit der aktuellen Forschungslage zu diesem Thema. Es wird eine systematische Recherche der Forschungsergebnisse anhand von klinischen und experimentellen Studien dargelegt; daraus werden erste Rückschlüsse für die Praxis gezogen.

### Das Beobachtungslernen und seine mögliche Rolle beim Placeboeffekt

Soziales Lernen wurde von Albert Bandura in seinen Arbeiten zur sozialen Lerntheorie analysiert [3, 12]. Während soziales Lernen den gesamten sozialen Kontext umfasst [15], bezieht sich Beobachtungslernen auf die Beobachtung einer Handlung an einer Modellperson und die darauf folgende Ausführung der Handlung durch den:die Beobachter:in selbst [4]. Der:die Beobachter:in muss die Handlung und ihren Sinn hierbei verstehen und setzt aktiv die aktuelle Lernerfahrung mit den aktuellen Kontextfaktoren und eigenen Vorerfahrungen zusammen. Hier müssen vier Voraussetzungen erfüllt sein [14]:

- Die Aufmerksamkeit der Beobachter:innen muss auf das Modell und dessen Handlung gerichtet sein.
- Die Beobachtung muss ins Gedächtnis überführt werden.
- Die Beobachter:innen müssen intellektuell und physisch in der Lage sein, die Handlung auszuführen.
- Die Beobachter:innen müssen motiviert sein, die Handlung durchzuführen.

Sind diese Voraussetzungen erfüllt, kann das Beobachtungslernen theoretisch die Erwartung beeinflussen und damit einen Einfluss auf den Placeboeffekt haben. Auch in der klinischen Praxis berichten Patient:innen von einem großen Einfluss durch die Beobachtung von Behandlungseffekten bei anderen Menschen, z. B. Verwandten oder Mitpatient:innen, auf die Erwartungen des eigenen Behandlungsergebnisses. Daraus ergeben sich für diese Arbeit zwei Fragen:

1. Kann Beobachtungslernen einen signifikanten Placeboeffekt auslösen?
2. Welche Einflussfaktoren auf dieses Beobachtungslernen konnten bisher identifiziert werden?

Zuerst werden die Ergebnisse allgemein zur Induktion des Placeboeffekts durch Beobachtungslernen und danach die Ergebnisse zu den Einflussfaktoren dargestellt. Eine tabellarische Auflistung der Ergebnisse findet sich in Tab. 1.

**Table 1:** 

Studie	Placeboeffekt	Lernintention Beobachtungslernen	Durchschnittliche Differenz	Effektstärke (d)	Modell
Bajcar et al. 2020 [2]	Ja	Modell für Durchführung des Experiments	0,69	0, 97 (groß), 0,74 (medium)	Live
Colloca et al. 2009 [9]	Ja	Modell für Durchführung des Experiments	1,92	n. a.	Live
Raghuraman et al. 2019 [18]	Ja	Explizite Instruktion	0,64^a^	0,36 (klein)	Bilder
Egorova et al. 2015 [11]	Ja	Explizite Instruktion	n. a.	n. a.	Video
Hunter et al. 2014 [15]	Ja	Modell für Durchführung des Experiments	1,8 (Gruppe 1), 2,1 (Gruppe 2)	1,46 (groß), 1,58 (groß)	Live, Video
Schenk et al. 2020 [20]	Ja	Explizite Instruktion	n. a.	n. a.	Video
Schwartz et. al. 2021 [22]	Nein (analgetisch), Ja (Funktionskapazität)	Coverstory	n. a.	n. a.	Live
Swider et al. 2013 [24]	Nein	Coverstory	n. a.	n. a.	Live
Swider et al. 2016 [25]	Ja	Coverstory	0,83	n. a.	Live
Tu et al. 2019 [26]	Ja	Explizite Instruktion	n. a.	n. a.	Video
Van Lierde et al. 2020 [27]	Nein	n. a.	n. a.	n. a.	Video
Zhang et al. 2017 [28]	n. a.	Explizite Instruktion	n. a.	n. a.	Video

^a^ auf Skala von 0–10 umgerechnet von 0–100

*n. a.* nicht angegeben

## Vorgehen bei der Studienauswahl

In diesem systematischen Review werden nur Studien einbezogen, die auf einer direkten Beobachtung, Beobachtung durch Videoaufnahmen oder Bilder der Behandlungseffekte eines Modells basieren. Studien, in denen nur verbale soziale Information ohne eine Beobachtungssituation vermittelt wird, wurden ausgeschlossen. Aus den oben genannten Fragestellungen ergeben sich die Schlagworte für die Literaturrecherche. In diesem Review ist der Placeboeffekt definiert als der Unterschied zwischen den Placebo-Cues, die mit einem niedrigen Schmerzrating in der Lernphase gepaart waren, und den Cues, die mit einem höheren Schmerzrating in der Lernphase gepaart waren.

Die folgenden Schlagwörter wurden verwendet, um die Datenbanken PsyIndex, APA PsycInfo, PsycArticles, PubMed und Web of Science zu durchsuchen: social learning/observational learning AND placebo AND pain/analgesia/hypoalgesia. Die Suche wurde am 01.05.2021 abgeschlossen. Die Suche ergab nach der Entfernung von Duplikaten 173 Ergebnisse. Nach der Anwendung der Ein- und Ausschlusskriterien (siehe Tab. 2) blieben 12 Studien übrig.

**Table 2:** 

Studienpopulation	Personen mit und ohne chronische Schmerzstörung
Intervention	Beobachtungslernen von Placebohypoalgesie
Vergleich	Differenz im Schmerzrating zwischen Placebo-Cues und neutralen Cues
Kontext	Labor- und klinische Studien
Studientyp	Experimentell und kontrolliert
Messwerte	Schmerz oder schmerzabhängige Variablen
Ausschluss	Nur soziale Kontextinformationen, Nocebostudien

## Induktion des Placeboeffekts durch Beobachtungslernen

### Operationalisierung des Beobachtungslernens von Placeboeffekten

**Figure 1:**
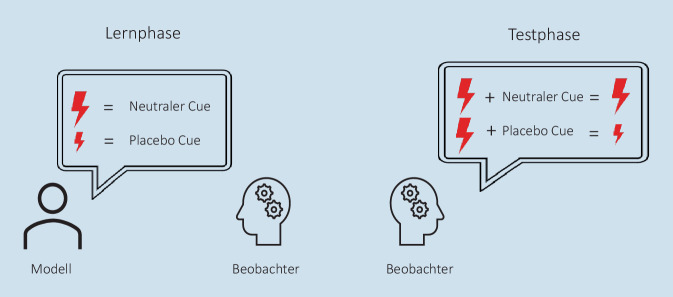

Fast alle Studien, die den Ein- und Ausschlusskriterien entsprachen [2, 9, 11, 15, 18, 20, 22, 24–27], verwendeten das folgende Modell für das Beobachtungslernen: Die Beobachter:innen sahen in einer Lernphase ein Modell, das nach einem bestimmten Hinweisreiz (Placebo-Cue) einen Schmerzreiz niedriger einstufte als nach einem anderen Hinweisreiz (neutraler Cue). In der Testphase wurden den Beobachter:innen Schmerzreize verabreicht, die sich in der Intensität nicht danach unterschieden, welcher Hinweisreiz dargeboten wurde (siehe Abb. 1). Die Schmerzratings nach dem Placebo-Cue und dem neutralen Cue wurden verglichen.

Von den zwölf Studien zeigten neun einen signifikanten Placeboeffekt durch das Beobachtungslernen

Die Arbeitsgruppe von Zhang et al. [28] verwendete ein etwas anderes Lernparadigma. Hier wurde wie oben beschrieben eine Lernphase mit Beobachtungslernen verwendet. Es wurde nun einige Tage später eine klassische Konditionierung durchgeführt. Hierbei zeigte sich, dass das Beobachtungslernen einige Tage zuvor einen signifikanten Einfluss auf die Größe eines Placeboeffekts hatte. Von den 12 einbezogenen Studien konnten neun [2, 9, 11, 15, 18, 20, 22, 24, 25] einen signifikanten Placeboeffekt durch das Beobachtungslernen zeigen (siehe Tab. 1). Dies scheint ein deutlicher Hinweis zu sein, dass Beobachtungslernen von Placeboeffekten unter Laborbedingungen möglich ist. In einer Studie der Arbeitsgruppe um Swider [24] wurde kein Placebo-, sondern nur einen Noceboeffekt gefunden. Van Lierde et al. [27] konnten zwar einen signifikanten Unterschied in der Erwartung durch die Lernphase feststellen, aber in der Testphase gab es keinen signifikanten Placeboeffekt. Ein großer Unterschied zwischen dem Design der anderen Studien und der Studie von Van Lierde et al. [27] ist, dass in diesem Design die Beobachter:innen die Handlung nur einmal sahen, während die anderen Studien mehrfach den Zusammenhang zwischen Placebo-Cue und Schmerzrating beim Modell beobachten konnten. Weitere Studien müssen zeigen, ob eine einmalige Beobachtung zu einem Placebo Effekt führen kann.

Eine Voraussetzung für Beobachtungslernen ist, dass der:die Beobachter:in die Handlung versteht

Nur eine Studie verwendete ein klinisches Setting [22]. In dieser Studie an Patient:innen mit chronischen Rückenschmerzen [22] sollte die Wirkung einer Verum-Medikation durch den sogenannten additiven Placeboeffekt (siehe Tab. 2), ausgelöst durch Beobachtungslernen, verstärkt werden. In dieser Studie zeigte sich ein signifikanter Placeboeffekt in der körperlichen Funktionskapazität, nicht aber in der durchschnittlichen Schmerzstärke der chronischen Schmerzen. In diesem klinischen Setting wurde die Verum-Medikation als Placebo-Cue verwendet. Das Beobachtungslernen fand bei einer zufälligen Begegnung mit dem Modell, einem vermeintlichen Patienten, während eines Routinearztbesuchs statt. Dieser berichtete von weniger Schmerzen und einer verbesserten Beweglichkeit durch die Verum-Medikation. Die verbesserte Beweglichkeit führte er den Patient:innen vor. Als Placeboeffekt wurde der Vergleich der durchschnittlichen Schmerzstärke unter der Verum-Medikation vor und nach dieser definiert. Es handelt sich also um einen Vorher-nachher-Vergleich, während bei den o. g. Studien zwei Bedingungen untersucht wurden, die in einer Lernphase gleichzeitig auftraten (neutrale Cues vs. Placebo-Cues). Das Modell zeigte hier nicht den Schmerzgesichtsausdruck bei der Bedingung vor der Verum-Medikation und einen neutralen oder fröhlichen Gesichtsausdruck nach der Einnahme der Verum-Medikation. Aber das Modell hatte einen Schmerzgesichtsausdruck, während er sagte, dass eine Bewegung vor der Behandlung nicht möglich war (Bedingung ohne Verum-Medikation – neutraler Cue) und danach (Bedingung mit Verum-Medikation – Placebo-Cue) wieder möglich war, was er direkt demonstrierte. Die Autor:innen argumentieren, dass die fehlende direkte Beobachtung der Schmerzreduktion dazu führte, dass kein analgetischer Placeboeffekt gefunden wurde. Weitere Studien an Patient:innen sind dringend erforderlich, um diese Effekte zu replizieren und ihre klinischen Implikationen zu überprüfen. Eine Voraussetzung für Beobachtungslernen ist, dass der:die Beobachter:in die Handlung versteht. Dies bedeutet in diesem Kontext, dass der:die Beobachter:in erkennt, dass der Placebo-Cue zu einem niedrigeren Schmerzrating oder assoziierten Maßen führt als der neutrale Cue. Um dies umzusetzen, wurden in einigen Studien die Beobachter:innen direkt instruiert, die Verbindung zwischen Schmerzrating und Cue zu lernen [11, 18, 19, 26, 28]. In anderen Studien wurde dies nicht offengelegt, sondern das Modell führte die praktische Durchführung des Experiments vor [2, 9, 15, 22]. Einige Studien verwendeten Coverstorys, um die Anwesenheit des Modells zu erklären. So sollte eine eher natürliche Beobachtungssituation generiert werden, in der der:die Beobachter:in zufällig das Modell beobachtet. In der Arbeitsgruppe um Swider [24, 25] war das Modell schon anwesend und wurde vorgezogen, da die Person unter Zeitdruck war (siehe Tab. 3). Es wurde eine verdeckte Aufmerksamkeitslenkung durchgeführt, indem die Beobachter:innen die Farbe des Cues und das Schmerzrating aufschreiben sollten, um dem:der Studienleiter:in zu helfen. Bei Schwartz et al. [22] wurde ebenfalls eine Coverstory direkt durch das Modell eingeführt. Der vermeintliche Patient kam unter dem Vorwand, den:die Arzt:Ärztin zu suchen, in das Behandlungszimmer und zeigte dann die Verbesserung der Schmerzintensität und Beweglichkeit (Intervention Beobachtungslernen) bzw. gab nur einen Fragenbogen ab (Kontrollgruppe).

**Table 3:** 

Begriff	Erläuterung
Placeboeffekt	Schmerzlinderung nach der Einnahme von Medikamenten ohne spezifischen Wirkstoff, nach Scheineingriffen oder anderen Cues
Noceboeffekt	Schmerzverstärkung nach der Einnahme von Medikamenten ohne spezifischen Wirkstoff, nach Scheineingriffen oder anderen Cues
Cue	Hinweisreiz (z. B. Formen, Farben, Licht)
Placebo	Eine Tablette oder Injektion ohne pharmakologischen Inhaltsstoff. Zum Placebo gehören auch Scheineingriffe oder Cues wie Licht oder Elektroden
Verum-Medikation	Medikament mit einem pharmakologischen Inhaltsstoff
Additiver Placeboeffekt	Zusätzlicher Effekt zu einer Verum-Medikation, der durch Placebo Mechanismen ausgelöst wird
Klassische Konditionierung	Die Koppelung eines unkonditionierten Stimulus (z. B. Futternapf) und der unkonditionierten Reaktion (z. B. Speichelfluss) mit einem neutralen Stimulus (z. B. Glocke). Dies führt zu einer konditionierten Reaktion auf den nun konditionierten Stimulus (vorher neutraler Stimulus)
Verbale Instruktion/verbales Lernen	Informationsvermittlung durch verbale Mitteilungen (z. B. „Bei dem grünen Licht werden Sie weniger Schmerzen spüren.“)
Soziales Lernen	Lernen durch eine soziale Situation, was über die reine Informationsvermittlung hinausgeht und den gesamten sozialen Kontext einbezieht (z. B. Zeitungsartikel über das Joggingverhalten anderer)
Beobachtungslernen	Spezialform des sozialen Lernens, bei dem der:die Beobachter:in das Modell bei einer Handlung beobachtet und diese selbst ausführt

Alle drei Ansätze (explizite Instruktion, Modell für Durchführung des Experiments, Verwendung einer Coverstory) scheinen keinen Einfluss darauf zu haben, ob der analgetische Placeboeffekt ausgelöst werden konnte, oder auf die Effektstärke. Die Studienzahl ist jedoch noch zu klein, um eine endgültige Bewertung abzugeben. An dieser Stelle ist zu erwähnen, dass bei den drei Studien, die eine Coverstory verwendeten, eine Studie [24] einen Noceboeffekt ausgelöst hatte. In unserer eigenen Studie konnte zwar in der Placebogruppe „Beobachtungslernen“ eine Reduktion der Schmerzen gezeigt werden, diese wurde aber auch in der Kontrollgruppe aufgefunden. Ein im Vergleich zur Kontrollgruppe signifikanter Placeboeffekt zeigte sich aber in der körperlichen Funktionskapazität, die sich in der Placebogruppe nach der sozialen Beobachtung signifikant verbesserte [22]. Aber auch hier ist die Anzahl der Studien zu klein, um eine endgültige Aussage zu treffen. Der Vorteil einer Coverstory ist, dass neben der Erkenntnis, dass Beobachtungslernen unter Laborbedingungen möglich ist, eine etwas höhere Generalisierbarkeit erreicht wurde. Für weitere Studien sollte dieser Aspekt genau geplant werden. Eine zufällige, nicht bewusste Begegnung mit dem Modell oder eine Coverstory, die zu wenig Aufmerksamkeit auf die zu beobachtende Handlung lenkt, könnte im Vergleich zu einer direkten Instruktion zum Placeboeffekt das Beobachtungslernen unterdrücken.

## Einflussfaktoren auf das Beobachtungslernen

### Aufmerksamkeitslenkung

Im Einklang mit der von Bandura postulierten notwendigen Aufmerksamkeitslenkung auf die Handlung wurden die Teilnehmer:innen instruiert, bestimmte Parameter wie Anzahl bestimmter Hinweisreize oder die Schmerzratings während der Beobachtung zu notieren oder sich zu merken [2, 9, 15, 24, 25, 28]. Auch die oben erwähnte Instruktion, den Hinweisreiz mit dem Schmerzrating in Verbindung zu bringen, ist eine solche Aufmerksamkeitslenkung [11, 18, 19, 26, 28]. Van Lierde et al. [27] berichteten keine Aufmerksamkeitslenkungsaufgabe, aber die Änderung in der Erwartung ist ein Hinweis darauf, dass die Teilnehmer:innen ihre Aufmerksamkeit auf die Handlung gerichtet hatten. Bei Schwartz et al. [22] wurde auch keine direkte Aufmerksamkeitslenkung instruiert, da dies die Coverstory hätte beeinträchtigen können und die Autor:innen davon ausgingen, dass das Erleben eines Patienten:einer Patientin mit der gleichen Medikation für die Schmerzpatient:innen hoch relevant wäre. Bei weiteren Studien sollten solche Aufmerksamkeitslenkungen berücksichtigt werden, die auch im realen klinischen Kontext verwendet werden könnten, um damit die klinische Relevanz der Studien zu erhöhen.

### Verwendete Cues und Schmerzreize

In der bisherigen Forschung zum analgetischen Placeboeffekt und Beobachtungslernen wurden unterschiedliche Placebo-Cues verwendet: Farb-Cues [2, 9, 15, 24, 25, 27, 28], Salben (Farbe oder Auftragungsort [18, 19]), abstrakte Formen [11], Gesichter mit einem neutralen Gesichtsausdruck [26] oder eine bestehende Schmerzmedikation [22]. Swider [25] untersuchte, ob bestimmte Cues anderen als Placebo-Cues überlegen waren, und konnten keinen Unterschied zwischen den verschiedenen Arten der Cues (grün, rot, Kreis, abstrakte Form) finden. Auch scheinen die verwendeten Schmerzarten wie elektrischer Reiz [2, 9, 15, 24, 25, 28] oder Hitze [11, 18, 19, 26] keinen Unterschied zu machen. Nur die Verwendung von Kältereizen [27] konnte keinen Placeboeffekt auslösen, was aber möglicherweise an anderen o. g. Unterschieden im Design gelegen haben könnte. Die Studie von Schwartz et al. [22] ist die bisher einzige Studie, in der chronische Schmerzen von Patient:innen auf einen Medikamenten-Cue hin untersucht wurden und Placeboeffekte auf die körperliche Funktionskapazität erzielt werden konnten.

### Effektstärke

Von den Studien, die einen signifikanten analgetischen Placeboeffekt finden konnten, haben alle den Placeboeffekt nach der o. g. Definition berechnet, aber nicht alle haben die durchschnittliche Differenz oder die Effektstärke angegeben, da z. B. der Fokus auf einer Auswertung von Daten der Elektroenzephalografie (EEG) oder Magnetresonanztomografie (MRT) lag.

Von den Studien, die dazu Daten angegeben hatten [2, 9, 15, 18, 25], befand sich der durchschnittliche Unterschied in dem Schmerzrating von x̄ = 0,64 bis 2,1 auf einer Skala von 0–10 und die Effektstärke von klein (d = 0,361) bis groß (d = 1,58) [8] (siehe Tab. 1). Aufgrund der wenigen Datenpunkte und des großen Range kann man noch nicht ableiten, wie groß der erwartbare Placeboeffekt durch Beobachtungslernen ist. Die Übertragung von Effektstärken, die unter Laborbedingungen und mithilfe von experimentell verabreichten Schmerzreizen an Personen ohne chronische Schmerzerkrankungen erreicht wurden, auf klinische Schmerzen, ist zwar nur bedingt möglich. Da aber unter diesen Bedingungen sehr große Effektstärken erreicht wurden, kann davon ausgegangen werden, dass theoretisch klinisch signifikante Effekte durch Beobachtungslernen möglich sind.

### Vergleich Beobachtungslernen zu anderen Lernarten

In den vorliegenden Studien gibt es erste Vergleiche zu anderen Lernarten wie klassischer Konditionierung und Lernen durch Instruktion. In diesem Review wird der Prozess der Instruktion als eine Art des Lernens verstanden: Die Instruktion: „Dieser Placebo-Cue wird zu weniger Schmerzen führen“ ist im Sinne des verbalen Lernens die Vermittlung der Verbindung „Placebo Cue = Schmerzreduktion“, was eine Lernart darstellt. Colloca et al., Egorova et al. und Tu et al. [9, 11, 26] fanden keinen Unterschied in der Stärke des Placeboeffekts zwischen klassischer Konditionierung und Beobachtungslernen. Bei Colloca et al. [9] war der Effekt durch Beobachtungslernen signifikant stärker als der durch eine verbale Instruktion erlernte. In der Arbeit von Hunter et al. [15] konnte kein signifikanter Placeboeffekt durch eine Instruktion ausgelöst werden. Dabei ist aber zu beachten, dass die verbale Instruktion nur einmalig angewendet wurde. Eine Lernphase, die wiederholt die Information vermittelt, dass der Placebo-Cue zu einem niedrigeren Schmerzrating führt, hätte dagegen möglicherweise einen stärkeren Placeboeffekt ausgelöst. Der Vergleich von Beobachtungslernen und klassischer Konditionierung zeigt, dass beide Lernarten zu ähnlich starken Effekten unter Laborbedingungen führen können [9, 11, 26]. Eine einmalige verbale Instruktion hingegen scheint einen schwächeren Placeboeffekt auszulösen als Beobachtungslernen. Aber auch hier liegen erst wenige Studien vor und weitere Forschung ist nötig.

### Unterschied zwischen einem Live-Modell, einer Videoaufnahme und Bildern eines Modells

In einem klinischen Setting, aber auch im Labor, kann es sehr aufwendig sein, immer ein Live-Modell zu implementieren, wobei dies für experimentelle bzw. quasi-experimentelle Studien sinnvoll sein kann (vgl. [22]). Daher ist es wichtig zu wissen, ob Beobachtungslernen des Placeboeffekts auch über Videoaufnahmen möglich ist. Für andere Gebiete konnte nachgewiesen werden, dass Beobachtungslernen auch über Video umsetzbar ist [4]. Raghuraman et al. [18] konnten sogar zeigen, dass Bilder eines Modells (schmerzverzerrter vs. neutraler Gesichtsausdruck) für Beobachtungslernen ausreichen, was besonders gut für EEG- und MRT-Studien anwendbar wäre. Vier Studien haben ein Live-Modell verwendet [2, 9, 22, 25], vier haben Videos eines Modells angefertigt [11, 19, 26, 28] und konnten einen signifikanten Placeboeffekt durch Beobachtungslernen nachweisen (siehe Tab. 1). Swider et al. [24], die einen Noceboeffekt gefunden haben, haben auch ein Live-Modell verwendet, während Van Lierde et al. [27], die keinen Placeboeffekt fanden, ein Video verwendeten. In der Studie von Schwartz et al. wurde ein Live-Modell verwendet und lediglich ein Placeboeffekt in der Funktionskapazität gefunden [22]. Hunter et al. [15] zeigten in ihrer Studie, dass sowohl ein Live-Modell als auch eine Videoaufnahme eines Modells zu signifikanten Placeboeffekten führten, die sich nicht signifikant in ihrer Effektstärke unterschieden. Die Evidenz deutet stark darauf hin, dass sowohl ein Live-Modell als auch eine Videoaufnahme für das Beobachtungslernen eines Placeboeffekts ausreicht.

### Geschlecht des Modells

Schon früh zeigte sich, wie wichtig die Merkmale des Modells sind [3]. So konnte gezeigt werden, dass Beobachtungslernen einen größeren Effekt hatte, wenn Modell und Beobachter:in das gleiche Geschlecht hatten [7]. Die vorliegenden Studien wurden nicht alle im Hinblick auf diesen Gendereffekt ausgewertet. Studien, die einen anderen Fokus als Geschlechterunterschiede hatten, wurden teilweise nur mit Teilnehmer:innen eines Geschlechts durchgeführt, z. B. mit einem weiblichen Modell und weiblichen Teilnehmerinnen [15, 25, 26], oder einem männlichen Modell und weiblichen Teilnehmerinnen [9, 28]. Andere hatten weibliche und männliche Beobachter:innen und entweder ein männliches [19, 22] oder weibliches Modell [1, 27] oder für alle ein weibliches und ein männliches Modell [11]. Hier wurden keine Analysen nach Geschlechtszugehörigkeit berichtet. Raghuraman et al. [18] berichteten bei einem männlichen Modell keinen Unterschied in der Stärke des Placeboeffekts durch Beobachtungslernen bei männlichen versus weiblichen Beobachter:innen. Die Forschergruppe um Swider [24] untersuchte den Effekt der Geschlechtszugehörigkeit bei Modell und Beobachter:in und fanden, dass der Noceboeffekt größer war, wenn ein männliches Modell beobachtet wurde, und zwar bei männlichen und weiblichen Beobachter:innen. Jedoch wurde bei dieser Studie ein Nocebo- und kein Placeboeffekt gefunden, weshalb die Generalisierbarkeit auf den Placeboeffekt nur bedingt gegeben ist. Es bedarf weiterer Studien, um abschließend zu beurteilen, inwieweit das Geschlecht von Modell und Beobachter:innen eine Auswirkung hat.

### Erwartung

Die bisherige Placeboforschung legt zumeist ein Modell zugrunde, in dem Erwartung ein zentraler Mediator ist [21], aber z. B. bei Konditionierungseffekten nicht immer vollständig der Mediator den Placeboeffekt darstellt [6, 23]. Die Frage ist, ob auch beim Beobachtungslernen die Erwartung oder Veränderungen in der Erwartung den Placeboeffekt vorhersagen können.

Raghuraman et al. [18] und Schenk et al. [19] haben die Erwartung abgefragt und fanden, dass die Erwartung für die Schmerzintensität von Placebotrials signifikant niedriger war als für Kontrolltrials. Sie haben nicht angegeben, ob die Erwartungsratings mit den Schmerzratings korrelieren.

Van Lierde et al. [27] konnten zeigen, dass Beobachtungslernen in ihrer Studie zu einer niedriger erwarteten Schmerzstärke im Falle der Placebo-Cues im Vergleich zu den neutralen Cues führte, fand hier aber keine Korrelation der erwarteten Schmerzstärke mit der erlebten Schmerzstärke, wobei in dieser Studie auch kein signifikanter Placeboeffekt ausgelöst werden konnte. Auch Schwartz et al. fanden bei chronischen Rückenschmerzpatient:innen [22] keine Änderung der Erwartung und auch keine Korrelation der Erwartung mit der durchschnittlichen Schmerzstärke.

Zwar scheint Beobachtungslernen zu einer Änderung der erwarteten Schmerzstärke zu führen, aber es bleibt weiter unklar, ob diese Erwartungsänderung die tatsächlich erlebte Schmerzstärke hervorsagen kann.

### Empathie und andere Persönlichkeitseigenschaften als Einflussfaktoren

Die Studie von Colloca & Benedetti [9] ist die erste Studie, in der Beobachtungslernen von Placeboanalgesie gezeigt werden konnte [9]. Empathie korrelierte in dieser Studie positiv mit dem Ausmaß der durch Beobachtungslernen induzierten Placeboanalgesie. Hunter et. al [15]. verglichen ein Live-Modell mit einer Videoaufnahme und konnten nur in der Gruppe mit dem Live-Modell eine Korrelation mit Empathie zeigen.

Auch Swider & Babel fanden mit einem Live-Modell eine positive Korrelation der Höhe des Noceboeffekts mit Empathie, aber hier war der prädiktive Effekt klein (Delta R^2^ = 0,07). In einer Folgestudie der Arbeitsgruppe ([25], Live-Modell) konnte kein Zusammenhang von Empathie und Placeboanalgesie gefunden werden. Die Autor:innen argumentierten, dass in ihren Studien durch die o. g. Coverstory ein eher zufälliges Beobachtungslernen stattfand und deshalb Empathie beim Beobachtungslernen keine Rolle spielte. Eine andere Studie [2] verwendete für die Durchführung ihres Experiments ein ähnliches Design wie Colloca & Benedetti [9], nämlich ein Live-Modell für das Lernen am Modell. Sie fand keine Korrelation von Empathie und Placeboanalgesie, was eher dagegen spricht, dass die Verwendung einer Coverstory die Korrelation beeinflusst. Auch bei der Verwendung von Bildern eines Modells [18] konnte kein signifikanter Zusammenhang mit Empathie festgestellt werden. Schließlich zeigte sich auch in der Studie mit chronischen Schmerzpatient:innen kein Zusammenhang des Placeboeffekts mit Empathie[22].

Ein Zusammenhang zwischen Angst vor Schmerz [2, 25] oder Persönlichkeitseigenschaften und dem Ausmaß an Beobachtungslernen konnte ebenfalls nicht gezeigt werden [2]. Die vorliegenden Studien deuten eher daraufhin, dass Empathie, Angst vor Schmerz oder Persönlichkeitseigenschaften keinen großen Einfluss auf das Beobachtungslernen von Placeboeffekten haben. Für weitere Forschung ist besonders relevant, ob sich ähnliche Ergebnisse in klinischen Settings mit Patient:innen mit chronischen Schmerzerkrankungen finden lassen.

## Ausblick

Die vorliegende Evidenz liefert die Grundlage dafür, dass theoretisch klinisch signifikante Effekte möglich sind. Auch in einer ersten Studie mit Patient:innen mit chronischen Rückenschmerzen zeigten sich Placeboeffekte durch Beobachtungslernen, und zwar im Bereich der körperlichen Funktionskapazität. In diesem klinischen Bereich sind weitere Studien nötig, um die Aussagen auch auf chronische Schmerzpatient:innen zu beziehen. Die Evidenz für Einflussfaktoren auf das Beobachtungslernen ist bisher schwach. Aus der bisherigen Forschung zum Beobachtungslernen lässt sich ableiten, dass der soziale Status des Modells [17] eine wichtige Rolle spielen könnte, was weiter untersucht werden sollte. Klinsch relevante Anwendungsgebiete könnte zum Beispiel das Auslösen von positiven Behandlungserwartungen sein.

## Fazit für die Praxis

Die Anzahl der vorliegenden Studien lassen noch keine belastbaren Aussagen zu, geben aber erste Hinweise für die folgenden Annahmen:

- Beobachtungslernen von Placeboeffekten ist unter Laborbedingungen möglich.
- Eine Aufmerksamkeitslenkung auf das zu beobachtende Modell ist wichtig.
- Eine ungünstige Coverstory, die die Valenz der Handlung herabsetzt, kann den Effekt schwächen.
- Die Effektstärken des Ausmaßes des Beobachtungslernens von analgetischen Placeboeffekten zeigten sich in den dargestellten Studien von klein bis groß.
- Die Effekte von klassischer Konditionierung und Beobachtungslernen sind vergleichbar.
- Eine einmalige verbale Instruktion löst kleinere Effekte aus.
- Live-Modelle, Videoaufnahmen und Bilder lösen ähnliche Effekte aus.
- Möglicherweise lösen männliche Modelle unabhängig vom Geschlecht der Beobachter:in größere Effekte aus.
- Beobachtungslernen verändert die Erwartung.
- Empathie, Angst oder Persönlichkeitseigenschaften haben keinen relevanten Einfluss auf das Beobachtungslernen.
